# Genetic signal maximization using environmental regression

**DOI:** 10.1186/1753-6561-5-S9-S72

**Published:** 2011-11-29

**Authors:** Phillip E Melton, Jack W Kent, Thomas D Dyer, Laura Almasy, John Blangero

**Affiliations:** 1 Department of Genetics, Texas Biomedical Research Institute, PO Box 760549, San Antonio, TX 78253, USA

## Abstract

Joint analyses of correlated phenotypes in genetic epidemiology studies are common. However, these analyses primarily focus on genetic correlation between traits and do not take into account environmental correlation. We describe a method that optimizes the genetic signal by accounting for stochastic environmental noise through joint analysis of a discrete trait and a correlated quantitative marker. We conducted bivariate analyses where heritability and the environmental correlation between the discrete and quantitative traits were calculated using Genetic Analysis Workshop 17 (GAW17) family data. The resulting inverse value of the environmental correlation between these traits was then used to determine a new *β* coefficient for each quantitative trait and was constrained in a univariate model. We conducted genetic association tests on 7,087 nonsynonymous SNPs in three GAW17 family replicates for Affected status with the *β* coefficient fixed for three quantitative phenotypes and compared these to an association model where the *β* coefficient was allowed to vary. Bivariate environmental correlations were 0.64 (± 0.09) for Q1, 0.798 (± 0.076) for Q2, and −0.169 (± 0.18) for Q4. Heritability of Affected status improved in each univariate model where a constrained *β* coefficient was used to account for stochastic environmental effects. No genome-wide significant associations were identified for either method but we demonstrated that constraining *β* for covariates slightly improved the genetic signal for Affected status. This environmental regression approach allows for increased heritability when the *β* coefficient for a highly correlated quantitative covariate is constrained and increases the genetic signal for the discrete trait.

## Background

The current availability of groups of correlated phenotypes for several common complex chronic diseases can aid in the study of these traits by providing data beyond what is contained in the phenotypes individually. Several earlier statistical genetic studies have shown that when correlations between phenotypes are explicitly modeled, they provide greater power than that provided by univariate analyses of individual traits [1-6]. Joint analysis of traits can also improve the detection of quantitative trait loci, where the effect sizes are too small to be found in single-trait analyses, and it can also inform the investigation of pleiotropy and co-incident linkage. A commonly encountered situation, in which the potential benefits of bivariate analysis are appreciated, is that of a discrete disease trait and a correlated quantitative phenotype. A number of multifactorial diseases, for example, diabetes or hypertension, studied as discrete traits, are also highly correlated with quantitative traits, physiological risk factors, or other continuously distributed biological characteristics. Although quantitative traits may not be used explicitly in the definition of disease status, both classes of information are useful and mutually supportive. However, these analyses primarily focus on the genetic correlation between the traits and do not take into account how the environmental correlation between the phenotypes may be used. This is important because often in epidemiological genetic studies of complex phenotypes, quantitative traits that are significantly correlated with the disease phenotype are included as covariates in the analysis, and not accounting for the environmental correlation leads to a less than optimal genetic signal.

Toward this end, we present a novel statistical genetics regression method that accounts for a portion of the environmental component using the Genetic Analysis Workshop 17 (GAW17) 1000 Genomes Project simulated family data between the discrete trait (Affected) and quantitative phenotypes Q1, Q2, and Q4 over all 200 replicates. We conducted bivariate polygenic analyses of the discrete trait with each of the three quantitative traits. We then used the resulting data from these analyses to calculate a new *β* coefficient for the quantitative trait, which was constrained to this value in a univariate analysis of affection status with the quantitative phenotype included as a covariate. Next, we compared the resulting model to a univariate analysis of affection status in which the quantitative trait was included as an unmodified covariate. Finally, we conducted genetic association tests using measured genotype analysis for affection status with each quantitative phenotype included as a covariate in two models: (1) a model in which the *β* coefficient was allowed to fluctuate and (2) a model in which the *β* coefficient was constrained. We used only the first three replicates of the GAW17 family data set to determine whether this method maximized the genetic signal for the true known genetic variants.

## Methods

### Data description

The GAW17 family data set contains 697 individuals divided into 194 nuclear families in 8 pedigrees with 202 founders from the 1000 Genomes Project. These family data include 13,875 autosomal single-nucleotide polymorphisms (SNPs) from 3,205 genes, with 7,087 of these genotyped SNPs in 1,890 genes being nonsynonymous. Each of the 200 simulated data sets includes the following information for each individual: affection status, three continuous quantitative traits (Q1, Q2, and Q4), age, smoking status, and sex [7]. These analyses were done with knowledge of the GAW17 answers.

### Analytical methods: environmental regression and association analysis

For the environmental regression, we used maximum-likelihood methods, taking into account relationships among family members, to determine heritability for Affected status and each of the three quantitative traits in a bivariate polygenic model using the computer program Sequential Oligogenic Linkage Analysis Routines (SOLAR) [8]. This bivariate method investigates the relationship of two related phenotypes simultaneously and tests for shared or overlapping genetic and environmental components [4]. Affection status was analyzed using a liability threshold model. Covariates used in these analyses were Age, Sex, and Smoking for all three quantitative phenotypes (Q1, Q2, and Q4) over all 200 replicates. Because variance components are sensitive to kurtosis, we transformed all quantitative variables using an inverse normalization procedure available in SOLAR [8]. We then used the resulting data from this analysis to calculate a new *β* coefficient for the quantitative trait using the equation:(1)

where:(2)(3)

 is the heritability of the discrete trait,  is the heritability of the quantitative trait, and *ρ_e_* is the environmental correlation between the discrete trait and the quantitative trait.

Next, we used the inverse of the resulting value as the *β* coefficient for the quantitative trait and constrained it in a univariate polygenic model, using the discrete phenotype as the trait and the quantitative variable as a covariate. This resulted in a new heritability, calculated by:(4)

where *β_e_* is the constrained *β* coefficient, *ρ_g_* is the genetic correlation between traits, *h_Q_* is the square root of , and *h_D_* is the square root of . We then compared the resulting heritability to a univariate polygenic model in which the quantitative trait was included as a covariate and the *β* coefficient was allowed to fluctuate.

For the association analysis we conducted a measured genotype analysis on each of the 7,087 nonsynonymous SNPs to calculate a nominal *p*-value for association using Affected status as the trait and Age, Sex, and Smoking as the covariates. To control for potential population stratification, we performed principal components analysis on genotype scores for 6,178 polymorphic synonymous SNPs in the 202 founders using the prcomp routine available in the R statistical package (http://www.r-project.org) [9]. We included the first four principal components (PC1–PC4) as covariates.

We conducted measured genotype analysis for each polymorphic SNP. The number of minor alleles was added to the quantitative polygenic genetic model as a covariate to assess the effect of the SNP genotype on the mean of the trait. This model was fitted to the data and compared with the null model of no difference in trait mean by genotype using a likelihood-ratio test. Twice the difference in log-likelihoods of these models was distributed as a chi-square random variable with 1 degree of freedom. The resulting likelihood-ratio test statistic was recorded for each nonsynonymous SNP. We then repeated this measured genotype association analysis with the *β* coefficient for the quantitative trait constrained in order to determine whether this method improved power to detect association. Because of issues of multiple testing, we initially used a highly conservative Bonferroni correction of 7.05 × 10^−6^ for genome-wide significance based on the GAW17 family data. However, as a result of linkage disequilibrium patterns in these data, it is likely that the total number of effective SNPs is lower than the total 7,087 SNPs used in the study. Therefore we calculated the effective number of SNPs using Moskvina and Schmidt’s method implemented in SOLAR [8,10]. We used the nominal *p*-value and the effective number of SNPs to determine an adjusted *p*-value for each gene to be used in multiple testing. This *p*-value was calculated using the equation:(5)

where “corrected” is the corrected *p*-value, “nominal” is the uncorrected *p*-value, and “effective” is the effective number of SNPs. This approach allows for nonindependence among family members and accounts for effects of other potential covariates [11].

## Results and discussion

For the environmental regression analysis, Table 1 shows the results of the three bivariate polygenic models conducted for this study across all 200 replicates. The highest heritability (0.6274) was for Q4, and the lowest heritability (0.3752) was for Q2. The highest environmental correlation with affection status was found for Q2 (0.7984), and the lowest environmental correlation was for Q4 (−0.1687). The direction of the sign for Q4 fits with the simulating model, in that Q4 is protective against Affected status.

**Table 1:** Bivariate models used for calculating the *β* coefficient for environmental regression

Bivariate model	Heritability (Affected) (SD)	Heritability (quantitative trait) (SD)	*ρ_g_*	*ρ_e_*
Affected * Q1	0.4383 (0.123)	0.5865 (0.058)	0.7673	0.6474
Affected * Q2	0.4848 (0.117)	0.3752 (0.071)	0.7091	0.7984
Affected * Q4	0.5116 (0.133)	0.6274 (0.067)	−0.2816	−0.1687

Values are averages across 200 GAW17 family replicates. SD is standard deviation. *ρ_g_* is the genetic correlation, and *ρ_e_* is the environmental correlation.

**Figure 1:**
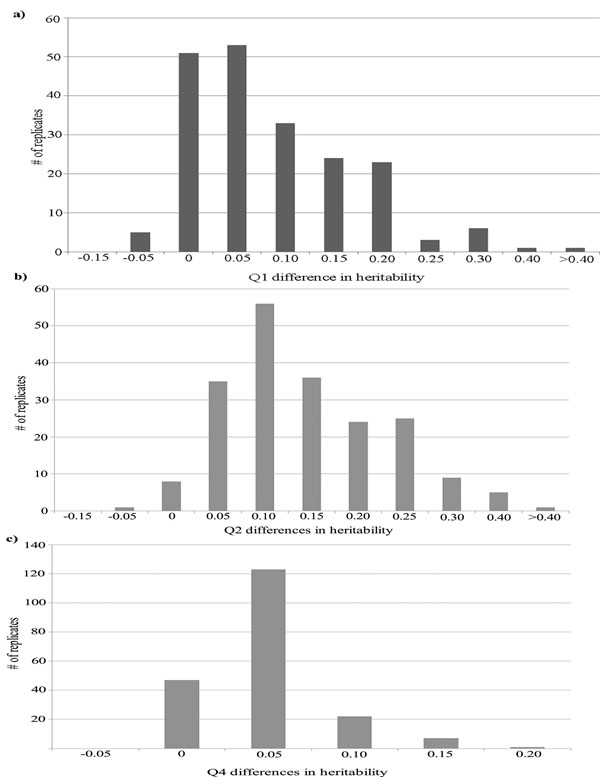
**Differences in heritability for 200 GAW17 family data replicates using affection status with quantitative phenotypes added as a covariate where the *β* coefficient is constrained for the quantitative trait versus where the *β* coefficient is allowed to fluctuate in a polygenic model.** (a) Phenotype Q1 (average difference = 0.061 [± 0.08]); (b) Q2 (average difference = 0.118 [± 0.085]); and (c) Q4 (average difference = 0.022 [± 0.033]).

The *β* coefficient was calculated for all three quantitative phenotypes (Q1, Q2, and Q4), and the inverse value was constrained in a univariate polygenic model, for affection status, as a covariate along with Age, Sex, and Smoking. This was then compared to a polygenic model for affection status where the quantitative phenotype was not constrained but included as a covariate. Overall, constraining the *β* coefficient improved the heritability of affection status through accounting for environmental correlation with each of the three quantitative phenotypes. Q2 demonstrated the greatest change in heritability for affection status with an average increase of 0.118 (Figure 1b). Q1 had an average increased change in heritability of 0.061 (Figure 1a). Q4 had the smallest increase in heritability with an average change of 0.022 (Figure 1c). The greatest improvement in heritability was seen for the quantitative trait covariate with the strongest environmental correlation. In this particular case, we used the inverse value of the environmental correlation, because the affection status was known, and defined it as Q1 + Q2 − Q4. By changing the sign, we are correctly accounting for the direction of effect in these data, given the implementation of the liability threshold model where a negative *β* coefficient implies a higher disease risk.

We conducted the genetic association analysis on affection status adjusting for all three quantitative phenotypes in the first three replicates of the data set. We compared a measured genotype association analysis of affection status in which the *β* coefficient for each quantitative phenotype was allowed to fluctuate to a measured genotype analysis in which the *β* coefficient was constrained to a fixed value in the first three GAW17 replicates. Of the 162 “true” genetic variants for the four GAW17 phenotypes, 58 (12 for Affected, 17 for Q1, 29 for Q2, and none for Q4) were found in these family data.

No genetic association that met our conservative criteria for genome-wide significance was detected for either the free association or the environmental regression association model. This lack of significant association may be due to the small sample size and the limited number of “true” Affected variants (12 SNPs) within these family data. Therefore we investigated the average differences in chi-square values for association tests in three different data sets under the assumption that an improvement in heritability would improve the genetic signal associated with affection status while decreasing the overall signal for nontrue variants (Table 2). The first of these data sets investigated chi-square differences in all 7,087 nonsynonymous SNPs available in the family data. The second looked at chi-square differences in the 58 “true” SNPs. The final model investigated chi-square variation in the 12 “true” Affected status variants. In replicate 3, where we detected the greatest increase in heritability with a constrained *β* coefficient, we also found a slight increase in the average chi-square differences (+0.171 for Q1, +0.334 for Q2) for SNPs truly associated with affection status in these family data. Although there is only a slight increase in chi-square, there are only 12 SNPs (C1S11396, C2S2288, C1S3181, C14S3704, C14S3706, C1S9189, C1S9266, C1S9445, C1S9455, C18S2492, C17S4578, and C19S4929) that are truly associated with affection status in the GAW17 family data. Of these 12 SNPs, only 5 (C14S3704, C14S3706, C1S9189, C1S9266, C17S4578) are present in more than five individuals, indicating that we may have limited power to accurately detect genome-wide significance in these data.

**Table 2:** Differences (Δ) in heritability and Χ^2^ for the first – replicates in the GAW17 data

Replicate	ΔH2R^a^ Q1	ΔQ1 chi-square, all (*n* = 7,087)	ΔQ1 chi-square, true (*n* = 58)	ΔQ1 chi-square, Affected (*n* = 12)	ΔH2R^a^ Q2	ΔQ2 chi-square, all (*n* = 7,087)	ΔQ2 chi-square, true (*n* = 58)	ΔQ2 chi-square, Affected (*n* = 12)	ΔH2R^a^ Q4	ΔQ4 chi-square, all (*n* = 7,087)	ΔQ4 chi-square, true (*n* = 58)	ΔQ4 chi-square, Affected (*n* = 12)
1	−0.01	−0.182	−0.728	−0.368	0.07	−0.205	−0.335	0.028	−0.012	0.504	0.041	0.096
2	−0.014	−0.107	−0.335	0.028	0.004	−0.198	−0.028	0.0003	0.095	0.012	0.005	−0.005
3	0.154	−0.132	−0.262	0.171	0.241	−0.302	−0.255	0.334	0.018	−0.038	−0.083	−0.099

^a^ Differences in heritability between a measured genotype association model in which the *β* coefficient is allowed to vary and the model in which the *β* coefficient is constrained to the environmental correlation between Affected status and a quantitative trait.

*n* is the number of SNPs in each set tested.

## Conclusions

As next-generation sequencing data become more available, an important consideration will be to maximize the ability to detect rare variants that have a large effect on chronic disease. The easiest way to detect these rare variants will be through large pedigrees, because rare variants will be amplified in families. Our results suggest that by controlling for some of the stochastic environmental noise between two highly correlated traits, we can improve the ability to identify genetic variants in pedigrees through an increase in heritability. For the current study, we proceeded with a two-step process. The first step was to conduct a bivariate polygenic analysis between a discrete trait and a quantitative trait to calculate a *β* coefficient for our quantitative trait. We found that this increased our average heritability when the *β* coefficient for the quantitative trait was constrained. We then compared two measured genotype association analyses, one in which the *β* coefficient was constrained for the quantitative trait to account for environmental correlations and the other in which the *β* coefficient was allowed to vary. Neither method identified any true associated variants in the GAW17 family dataset when a strict correction for multiple testing was used, but the novel environmental regression method did allow for an increase in the chi-square value for SNPs known to be associated with affection status, particularly in replicates in which the heritability was improved.

## Competing interests

The authors declare that there are no competing interests.

## Authors’ contributions

PEM carried out the statistical analysis, participated in the study design and drafted the manuscript. JWK aided in the statistical analysis and participated in the study design. TDD participated in the study design and statistical analysis. LA aided in the study design and helped draft the manuscript. JB conceived of the study and participated in its design and coordination.
